# Impact of Dialectical Behavior Therapy Prolonged Exposure protocol on trauma-related symptoms in Egyptian women with Borderline Personality Disorder

**DOI:** 10.1192/j.eurpsy.2022.1926

**Published:** 2022-09-01

**Authors:** M. Esmaiel, M. Abdelhamied

**Affiliations:** Faculty of Arts - Alexandria University, Psychology, Alexandria, Egypt

**Keywords:** Dialectical Behavior Therapy Prolonged Exposure (DBT PE) Protocol, Trauma - related symptoms, borderline personality disorder

## Abstract

**Introduction:**

Although around 50% of individuals with borderline personality disorder (BPD) suffer from trauma-related disorders, literature lacks a specific treatment for these serious co-occurring problems. Dialectical Behavior Therapy Prolonged Exposure (DBT PE) is a recent protocol developed by Melanie Harned, integrating (PE) into standard (DBT). The protocol has showed promising results in treating comorbid PTSD in BPD patients. The current study, however, was the first trial to apply DBT PE protocol in Egypt.

**Objectives:**

To investigate the efficacy of (DBT PE) protocol in reducing trauma-related symptoms (psychological trauma symptoms and trauma-related cognitions) among Egyptian women with BPD.

**Methods:**

Sixteen women diagnosed with BPD and trauma-related symptoms, were recruited from “DBT clinic”, a private outpatient clinic in Alexandria, Egypt and randomly divided into equivalent (Therapeutic & Control) groups. The therapeutic group received DBT PE protocol while the control group received Treatment as usual (TAU). Participants were assessed pre and post-intervention using: The short version of the Borderline Symptom list, The Trauma Symptom Checklist-40, and The Posttraumatic Cognitions Inventory. The therapeutic group started treatment with standard comprehensive DBT concurrently with DBT PE protocol (14 individual sessions, 120 minutes/ week), according to readiness criteria suggested by the treatment developer.

**Results:**

Patients who received DBT PE protocol showed significantly lower degrees of psychological trauma symptoms and trauma-related cognitions compared to patients in control group.

**Conclusions:**

Despite being applied for the first time in Egypt, DBT PE protocol proved to be an effective intervention in reducing trauma-related symptoms in a sample of Egyptian BPD patients without any need to modify the original protocol.

**Disclosure:**

No significant relationships.

